# Non-Contact Measurement of Human Respiration and Heartbeat Using W-band Doppler Radar Sensor

**DOI:** 10.3390/s20185209

**Published:** 2020-09-12

**Authors:** Heesoo Kim, Jinho Jeong

**Affiliations:** Department of Electronic Engineering, Sogang University, 35 Baekbeom-ro, Mapo-gu, Seoul 04107, Korea; 0167124@naver.com

**Keywords:** doppler, millimeter-wave, radar, sensor, vital sign

## Abstract

This paper presents a W-band continuous-wave (CW) Doppler radar sensor for non-contact measurement of human respiration and heartbeat. The very short wavelength of the W-band signal allows a high-precision detection of the displacement of the chest surface by the heartbeat as well as respiration. The CW signal at 94 GHz is transmitted through a high-gain horn antenna to the human chest at a distance of 1 m. The phase-modulated reflection signal is down-converted to the baseband by the quadrature mixer with an excellent amplitude and phase matches between I and Q channels, which makes the IQ mismatch correction in the digital domain unnecessary. The baseband I and Q data are digitized using data acquisition (DAQ) board. The arctangent demodulation with automatic phase unwrapping is applied to the low-pass filtered I and Q data to effectively solve the null point problem. A slow-varying DC component is rejected in the demodulated signal by the trend removal algorithm. Then, the respiration signal with a frequency of 0.27 Hz and a displacement of ~6.1 mm is retrieved by applying a low-pass filter. Finally, the respiration signal is removed by the band-pass filter and the heartbeat signal is extracted, showing a frequency of 1.35 Hz and a displacement of ~0.26 mm. The extracted respiration and heartbeat rates are very close to the manual measurement results. The demonstrated W-band CW radar sensors can be easily applied to find the angular location of the human body by using a phased array under a compact size.

## 1. Introduction

Continuous-wave (CW) radar sensors based on Doppler effect have been widely used to remotely detect human vital signs such as respiration and heartbeat rates [1,2]. The CW signal experiences the phase modulation by the respiration and heartbeat motion when it is reflected from human body. Then, the radar sensor extracts the rates and displacements of the respiration and heartbeat from the received signal. Most of the reported CW radar sensors for vital sign detection are implemented using low microwave frequency such as 915 MHz, 2.45 GHz, and 5.8 GHz, at which the electromagnetic waves exhibit low attenuation in human tissues as well as in free space [1,3]. Therefore, the signal reflected from the chest surface and even the heart is strong enough for the radar sensors to detect the respiration and heartbeat rates. However, the low microwave frequency requires large-size components and exhibits relatively low resolution without the location information of the human body due to its long wavelength. 

As the carrier frequency of the radar sensors increases, the phase of the reflected signal is more sensitively modulated by the displacement due to a short wavelength, allowing higher accuracy in the detection of vital signs [2]. In addition, at high frequency-like millimeter-waves, the high-gain antenna or beam-forming array can be more easily implemented in a more compact size [2]. Therefore, millimeter-wave CW radar sensors can provide the angular location of the human body, as well as vital signs, if the beam-forming techniques are combined. However, it is known that millimeter-waves are not suited for the detection of the human heartbeat, because of a significant attenuation of millimeter-wave in human tissues [1]. 

In general, the human chest surface moves back and forth due to respiration, with a displacement ranging from 4 to 12 mm [4] at a rate of 0.2–0.34 Hz [5]. It is also known that the heartbeat vibration can be propagated to the chest surface, generating a chest wall displacement of 0.2–0.5 mm [6] at a rate of 1–1.34 Hz [5,7,8]. That is, this displacement by the heartbeat is much less than that by respiration. Therefore, it is difficult to detect the heartbeat signal by using a radar sensor with a low carrier frequency. A millimeter-wave CW radar sensor with a shorter wavelength is more suited for this application. 

In this paper, we applied the W-band frequency (94 GHz) for non-contact detection of human respiration and heartbeat. For this purpose, the W-band CW radar sensor was implemented in waveguide-based modules using horn antenna, quadrature mixer module, low noise amplifier (LNA) module, waveguide components, and frequency multiplier [9]. The reflected signals from the human chest containing the displacement information were down-converted into the baseband and digitized for the digital signal processing, such as low-pass filtering, arctangent demodulation, trend and peak removal, and band-pass filtering, to accurately extract the respiration and heartbeat signals. Section 2 discusses the operating principle of the designed W-band CW radar sensor including the detection algorithms. The results of the measurements of human respiration and heartbeat are presented in Section 3. 

## 2. Design of W-band CW Radar Sensor

### 2.1. Operation Principle 

**Figure 1:**
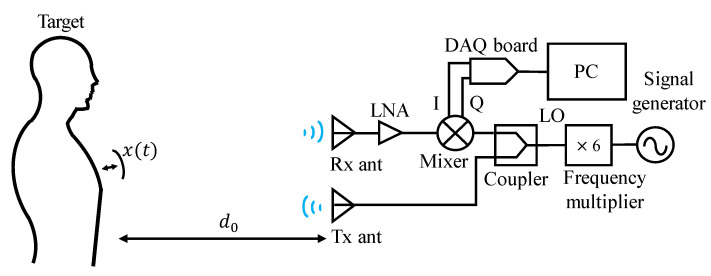
Block diagram of non-contact measurement of human vital sign using W-band continuous-wave (CW) radar.

Figure 1 shows the block diagram of the non-contact measurement of human vital signs using W-band CW Doppler radar, which is basically a direct-conversion transceiver operating at a carrier frequency of $f_{0}$ = 94 GHz [10]. The motion of human chest surface caused by respiration and the heartbeat is represented by the displacement x(t). The transmit signal $S_{tx}\left(t\right)$ is an unmodulated CW signal with amplitude A at a carrier frequency $f_{0}$ which is generated from the local oscillator (LO). Then, $S_{tx}\left(t\right)$ can be written as

(1) $$S_{tx}\left(t\right)=\;A\cos\left(2\pi f_{0}t\;+\;\theta \left(t\right)\right)$$

where θ(t) represents the phase noise of the LO. Then, the received signal $S_{rx}\left(t\right)$ which is reflected from the human chest surface at a distance $d_{0}$ from the radar can be represented with a reduced amplitude $A^{\prime }$ as

(2) $$S_{rx}\left(t\right)\;=A^{\prime }\cos\left(2\pi f_{0}\left(t\;-\;\Delta t\right)\;+\;\theta \left(t\;-\;\Delta t\right)\right)$$

A round-trip time Δt is given by $2\left(d_{0}\;+\;x\left(t\right)\right)/c$, where c is the speed of the electromagnetic wave in free space. Then, the baseband IQ signals are obtained by the quadrature mixer as follows.

(3a) $$I\left(t\right)\;=\;A_{b}\cos\left(\frac{4\pi }{\lambda }\left(d_{0}\;+\;x\left(t\right)\right)\;+\;\Delta \theta \left(t\right)\right),$$

(3b) $$Q\left(t\right)\;=\;A_{b}\sin\left(\frac{4\pi }{\lambda }\left(d_{0}\;+\;x\left(t\right)\right)\;+\;\Delta \theta \left(t\right)\right)$$

where Δθ(t) is a residual phase noise given by Δθ(t) = θ(t) −θ(t − Δt) and $\lambda \;=\;c/f_{0}$ is a wavelength. As shown in this equation, the displacement information is included in the phase of the I and Q signals in this CW radar. In particular, the displacement x(t) by human respiration and the heartbeat consists of very low-frequency components of less than a few Hz [5]. Unfortunately, the phase noise of the oscillators generally exhibits a 1/f dependency, implying a very high noise power at very low frequency [11]. Therefore, the phase noise can lead to severe errors in the detection of x(t), unless it is sufficiently suppressed. Note that the same oscillator is used for the transmit signal and the LO for quadrature mixer in the receiver as shown in Figure 1. This implies that the received and LO signals exhibit the same phase noise characteristics. In addition, the round-trip time is short enough in this short-range application that Δθ(t) = θ(t) − θ(t − Δt) in (3) is negligibly small. Thus, the errors caused by the phase noise of the local oscillators can be effectively mitigated by this range correlation effect [12]. For example, a round-trip time for the distance $d_{0}$ of 1 m is only 0.67 ns which is a sufficiently short time, considering the slowly varying phase noise with the frequency around human respiration and heartbeat rates (< 10 Hz). Therefore, it is fairly valid to assume Δθ(t)≅0 in the experiment.

In (3), we assume that there are no imbalances in the amplitude and phase of I and Q signals. However, there can exist imbalances in the real quadrature mixer caused by the mismatches in the circuits of I and Q channels which can generate DC offsets as well. Fortunately, it was shown in [13] that the quadrature mixer designed by the authors significantly reduced the imbalances at W-band. It is found in the experiment of vital sign detection that the mixer does not give rise to the errors associated with the imbalances. Therefore, there is no need for the algorithm to correct the IQ imbalance in the digital domain. 

The displacement x(t) can be obtained by the arctangent demodulation from the baseband IQ signals in (3), as described in (4). The argument φ(t) contains a DC value $\varphi_{0}=4\pi d_{0}/\lambda$ which can be easily eliminated in the digital domain. Then, x(t) is extracted from the variation in the phase Δφ(t), as given in (4b). This arctangent demodulation using both I and Q signals can effectively solve the null point problem which produces an inaccuracy in the vital sign detection at specific distances with a period of λ/4 [11].

(4a) $$\varphi \left(t\right)\;=\;\arctan\left[\frac{Q\left(t\right)}{I\left(t\right)}\right]\;≅\frac{4\pi }{\lambda }\left(d_{0}\;+\;x\left(t\right)\right)\;=\;\varphi_{0}\;+\;\Delta \varphi \left(t\right),$$

(4b) $$∴x\left(t\right)\;=\;\frac{\lambda }{4\pi }\Delta \varphi \left(t\right).$$

### 2.2. Extraction of Respiration and Heartbeat Signals 

**Figure 2:**
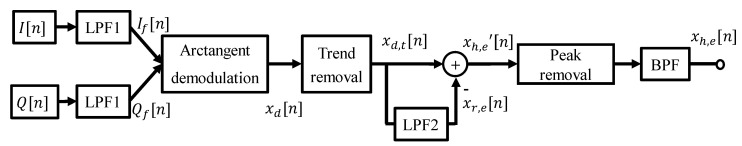
Digital signal processing for vital sign extraction.

The baseband IQ signals in (3) are digitized by the data acquisition (DAQ) board with a sampling frequency $f_{s}$ of 5 kHz. Figure 2 shows the block diagram demonstrating the procedure of displacement extraction from the digitized IQ signals using Matlab by Mathworks. They are first passed through the low-pass filter (LPF1) in order to reject the spurious signals, such as harmonics in I and Q signals or high-frequency noises including quantization noise of DAQ board. For this purpose, a moving average filter is utilized for LPF1 with a length of 333 ($f_{c}$ = 15 Hz).

The low-pass filtered IQ signals undergo the arctangent demodulation as given in (4). Unfortunately, it produces the phase discontinuity and distorts the displacement signal. In other words, the output of the arctangent function, Δφ(t), is wrapped between −π/2 and π/2, if |Δφ(t)| is greater than π/2. Note that |Δφ(t)|=π/2 corresponds to $x\left(t\right)=\frac{\lambda }{8}=0.4$ mm at $f_{0}=94$ GHz, which is smaller than the nominal displacement of the human chest surface by respiration [14]. Therefore, the phase discontinuity can occur very often in the arctangent demodulation in the millimeter-wave CW radars. In this work, an extended differentiate and cross-multiply (DACM) algorithm was utilized for the automatic correction of phase discontinuity, where the phase was calculated by the integration of the differentiated arctangent function in the discrete-time domain, as described in (5) [14].

(5a) $$\Delta \varphi \left[n\right]=\;\frac{I\left(n\right)\left\{Q\left[n\right]-Q\left[n-1\right]\right\}-Q\left(n\right)\left\{I\left[n\right]-I\left[n-1\right]\right\}}{I{\left[n\right]}^{2}+Q{\left[n\right]}^{2}},$$

(5b) $$∴\varphi \left[N\right]=\;\sum_{k=2}^{N}\frac{I\left[k\right]\left\{Q\left[k\right]-Q\left[k-1\right]\right\}-Q\left[k\right]\left\{I\left[k\right]-I\left[k-1\right]\right\}}{I{\left[k\right]}^{2}+Q{\left[k\right]}^{2}}.$$

Human random motion during the measurement can create a slow variation in $d_{0}$ (4a), which in turn produces the trend in the demodulated signal $x_{d}\left[n\right]$. The trend is removed by subtracting a DC component from $x_{d}\left[n\right]$ in every interval (2.9 s), where each interval overlaps half. The trend-removed signal $x_{d,t}\left[n\right]$ has the displacement components by both respiration and heartbeat. The respiration signal has a larger displacement at a lower rate than that of the heartbeat [4,5,6]. Therefore, it is difficult to extract the heartbeat signal from $x_{d,t}\left[n\right]$, since it may fall into the harmonics of the respiration signal. In this work, the displacement by respiration $x_{r,e}\left[n\right]$ was first extracted by low-pass filtering $x_{d,t}\left[n\right]$ with a moving average filter of length 1667 ($f_{c}$ = 3 Hz).

Then, it is removed from $x_{d,t}\left[n\right]$, that is, $x_{h,e}\prime \left[n\right]=x_{d,t}\left[n\right]-x_{r,e}\left[n\right]$ as illustrated in Figure 2. However, $x_{h,e}\prime \left[n\right]$ can include abnormal values generated by this subtraction, so that the peaks greater than the threshold are removed to reject abnormal values. The resulting signal is band-pass filtered to obtain the displacement by the heartbeat $x_{h,e}\left[n\right]$, using an elliptic filter with an order of 20 and a passband of 0.8-2 Hz. 

## 3. Measurement

### 3.1. Waveguide-Based W-band Doppler Radar

**Figure 3:**
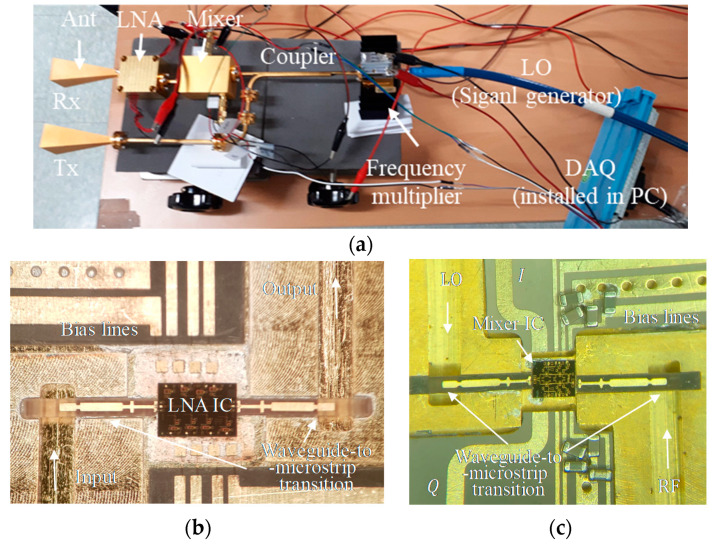
Fabricated W-band Doppler radar. (**a**) Overall view. (**b**) Waveguide low noise amplifier (LNA) module [15]. (**c**) Quadrature mixer module [13].

Figure 3 shows the implemented W-band Doppler radar using waveguide modules [9]. W-band CW signal is produced by the signal generator (8341A by Agilent) followed by the frequency multiplier-by-6 (AMC-10-RFHB0 by Millitech). Tx signal is radiated into the object through a horn antenna with a gain of 25 dBi. The same type of horn antenna is used as a receive antenna. Several waveguide bends and thru sections were employed in the transmitter to align Tx and Rx antennas. The high-gain antenna, which can be more easily implemented with a compact size at millimeter-wave frequency, is useful in reducing the artifacts by the reflection from the clutter around the object. In addition, its narrow beam-width can be beneficial in finding the angular location of the object. 

In the receiver, the LNA module was fabricated using a commercial W-band LNA IC and E-plane probe waveguide-to-microstrip transitions, as shown in Figure 3b. It exhibits a gain of 18.7 dB with noise figure of 4.4 dB at 94 GHz [15]. The coupler, the 3-dB power divider, enables the Tx signal to be used as the LO for the down-conversion mixer in the receiver. This implies that the received and LO signals exhibit the same phase noise characteristics. Therefore, the phase noise will be cancelled out by the mixer as explained in Section 2.1. The image rejection mixer, which was developed by the author, was used as a quadrature mixer without the off-chip IF 90° hybrid [13,16]. It showed very high image rejection ratio of ~40 dB with a conversion loss of ~11.6 dB. It was allowed by the significant reduction in the amplitude and phase imbalances between I and Q channels by using buffer amplifier control. The developed mixer IC was packaged using E-plane probe transitions as shown in Figure 3c. Two mixer output signals (I and Q) were then captured by the personal computer (PC) through a 12-bit data acquisition (DAQ) board (PCIE-1810-AE by Advantech). The performance of the radar components used in this work is summarized in Table 1.

### 3.2. Measurement of Human Respiration and Heartbeat

The implemented W-band radar sensor is used for vital sign detection as illustrated in Figure 1, where the distance $d_{o}$ = 1 m. The transmit CW power is 4.9 dBm at a carrier frequency $f_{o}$ = 94 GHz. The received signal is amplified by the LNA and down-converted by the mixer. The received power at the antenna is computed using the digitized mixer output voltages to be −41.8 dBm. Based on the radar equation, the radar cross section (RCS) of the human chest is estimated to be 0.042 $m^{2}$ which seems to be a reasonable value, compared to the reported RCS of $0.16{\;m}^{2}$ at 76 GHz in [17].

**Figure 4:**
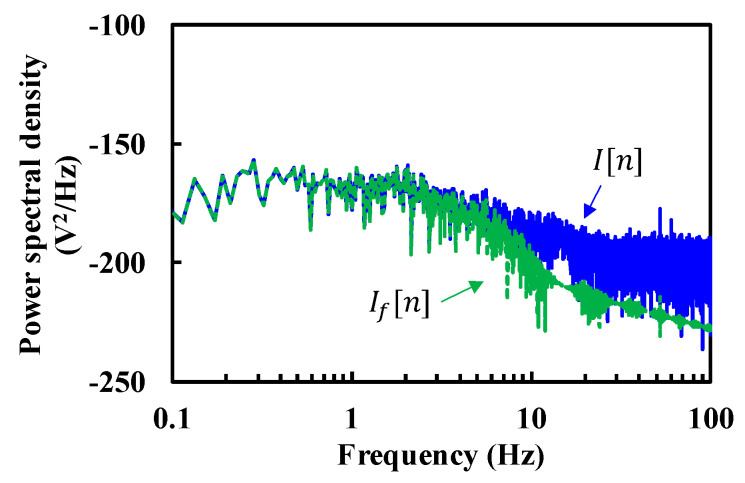
Power spectral density of I signal before and after LPF1 (I[n] and $I_{f}\left[n\right]$, respectively).

The respiration and heartbeat were measured for 52 s. Manual measurement shows the respiration of 14 breaths (0.27 Hz) and the heartbeat of 70 beats (1.35 Hz), respectively. Figure 4 shows the power spectral density of I signals before and after low-pass filtering (LPF1). It demonstrates that the high frequency noise was reduced by the LPF1. It also shows that the phase noise or 1/f noise of LO was significantly suppressed in I and Q signals by using Tx signal as the LO for the mixer. 

**Figure 5:**
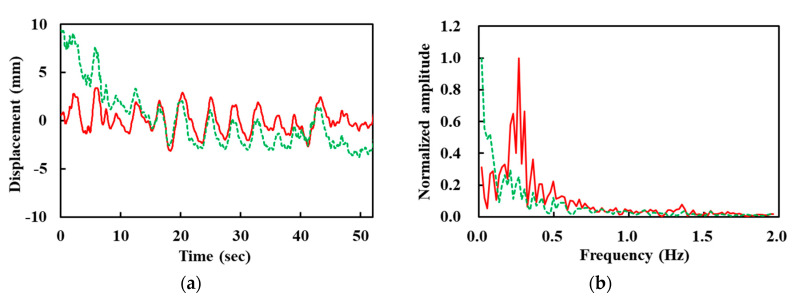
Demodulated ($x_{d}\left[n\right]$, dashed) and trend-removed signals ($x_{d,t}\left[n\right]$, solid). (**a**) Time-domain waveforms. (**b**) Normalized amplitude in frequency domain.

The filtered I and Q signals are used to retrieve the phase using arctangent demodulation. Figure 5a shows the demodulated signal ($x_{d}\left[n\right]$, dashed) which contains a slow-varing DC component, or trend. They are represented by a high DC-like component in the frequency domain shown in Figure 5b. Therefore, the trend removal algorithm was applied to suppress this component by each interval and obtain trend-free signals ($x_{d,t}\left[n\right]$, solid curves in Figure 5). We can easily recognize that both waveforms ($x_{d}\left[n\right]$ and $x_{d,t}\left[n\right]$) contain the respiration signal with a frequency of around 0.27 Hz. By contrast, the heartbeat signal is rarely noticeable in the time-domain waveform, because its amplitude is much lower than the harmonics of respiration and trends. 

**Figure 6:**
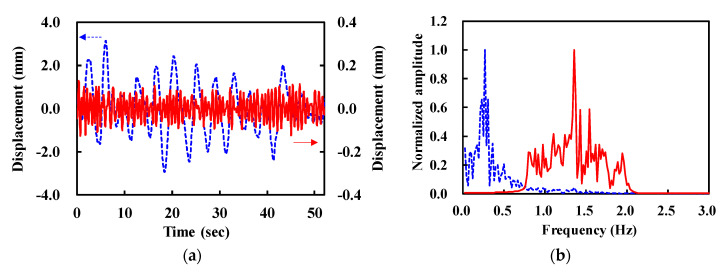
Extracted respiration ($x_{r,e}\left[n\right]$, dashed) and heartbeat ($x_{h,e}\left[n\right]$, solid) signal. (**a**) Time domain waveforms. (**b**) Normalized amplitude in frequency domain.

Figure 6 shows the extracted respiration and heartbeat signals, $x_{r,e}\left[n\right]$ and $x_{h,e}\left[n\right]$, respectively. It is found that the displacement by respiration and heartbeat is around 6.1 and 0.26 mm, respectively, which fall within the range reported in [4,6]. Thus, it can be stated that very tiny chestwall movement by the heartbeat can be detected by W-band Doppler radar. Figure 6b shows the frequency component of two signals. The peak amplitude was obtained at the frequency of 0.27 Hz for respiration and 1.35 Hz for the heartbeat, which are very close to manually-measured results. 

We also carried out the experiment on a person with different respiration and heartbeat rates (0.33 and 1.1 Hz, respectively) at the distances $d_{o}$ = 0.3 and 1.5 m. The same measurement setup and algorithm were used as in the previous experiment. The two measurements at the different distances exhibited different trends because of the random motions of the person, but the extracted respiration and heartbeat rates were very close to the manual measurement results in both distances. This result demonstrates that the measurement is not very sensitive to the distance and displacement.

### 3.3. Measurement Using Respiration Model

**Figure 7:**
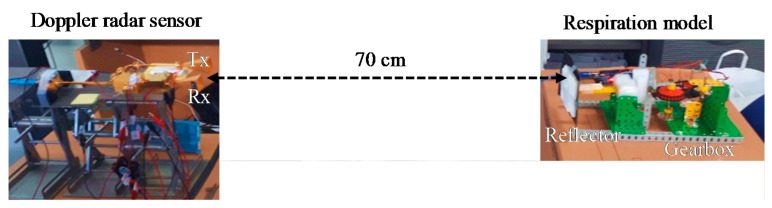
Respiration model experiment setup.

In order to further verify the operation of the designed W-band CW Doppler radar, the respiration model was developed as shown in Figure 7, where the reflector was designed to move back and forth to mimic human chest motion by respiration [14,18]. It was controlled to have a displacement of 20.0 mm at a rate of 0.5 Hz using the motor and microcontroller (Arduino Uno). The reflector was made of rubber and styrofoam with a size of $0.15\;\times 0.10{\;m}^{2}.$ The LPF1 with a length of 63 was used in this experiment, with the same LPF2 as in the previous experiment. The trend was removed for every 2 s.

**Figure 8:**
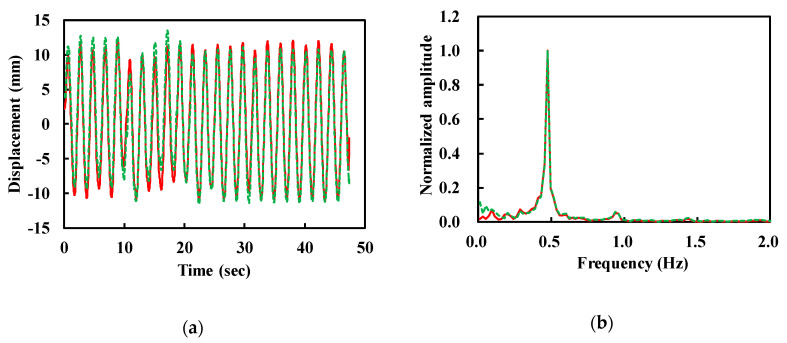
Demodulated ($x_{d}\left[n\right]$, dashed) and trend-removed signals ($x_{d,t}\left[n\right]$, solid). (**a**) Time domain waveforms. (**b**) Normalized amplitude in frequency domain.

The peak removal and band-pass filtering used for human vital sign detection were not performed in this experiment. Figure 8 shows the demodulated signals with and without trend removal. The trends are represented by the noise near DC in Figure 8b. In this experiment, the reflector was almost fixed during the measurement for 47 s. Therefore, there were no significant trends. The displacement was extracted to be 19.8 ± 2.1 mm with a rate of 0.48 Hz, which verifies the detection accuracy of the fabricated W-band radar sensor. 

## 4. Conclusions

In this paper, the W-band CW radar sensor was designed for non-contact detection of human respiration and heartbeat. It was implemented using waveguide-based modules such as horn antenna, LNA modules, quadrature mixer modules, frequency multiplier and waveguide components, to demonstrate the feasibility of W-band radar sensors for respiration and heartbeat measurement. The W-band CW signal was transmitted and reflected from the chest surface. Direct conversion was performed using Tx signal as LO for the quadrature mixer, which sufficiently suppressed the phase noise effect [11]. The quadrature mixer with minimized imbalances between I and Q channels made the IQ mismatch correction unnecessary and simplified the digital signal processing. The arctangent demodulation with automatic phase unwrapping was utilized to obtain the displacement signals without null-point issues. Together with the trend and peak removal, low-pass and band-pass filtering allows the accurate extraction of very small displacement by the heartbeat from very large respiration signals, thanks to the very short wavelength of W-band frequency. This work demonstrates that the millimeter-wave Doppler radar sensor can be used for the accurate detection of the human heartbeat as well as respiration. The radar components such as LNA, mixer, and frequency multiplier can be integrated in a single chip for the compact and low-cost implementation of the radars. The waveguide horn antenna can also be replaced with the compact planar antenna, such as microstrip patches which can be easily integrated onto the radar chip. The W-band radar demonstrated in this work can be applied for the detection of the location of the human body by adopting the phased array and other radar techniques, such as frequency-modulated CW radars. The short wavelength of millimeter-wave is very useful for the miniaturization of the radar sensors with beam-forming capability.

## Figures and Tables

**Table 1:** Performance of W-band radar components.

	LNA	Mixer	Antenna	Signal Generator	Frequency Multiplier
Gain (dB)	18.7	−11.6	25	-	8.6
NF (dB)	4.4	-	-	-	-
Phase noise (dBc/Hz)	-	-	-	−54, −60 *	-

* Offset frequency of 30 and 100 Hz, respectively, at a carrier frequency of 15.67 GHz.
